# Study on the In Silico Screening and Characterization, Inhibition Mechanisms, Zinc-Chelate Activity, and Stability of ACE-Inhibitory Peptides Identified in Naked Oat Bran Albumin Hydrolysates

**DOI:** 10.3390/foods12112268

**Published:** 2023-06-05

**Authors:** Yan Li, Junru Li, Chaoxia Cheng, Yajun Zheng, Hanxu Li, Zilin Zhu, Yuxiang Yan, Wenhui Hao, Nan Qin

**Affiliations:** 1 College of Food Science, Shanxi Normal University, Taiyuan 030092, China; liyan@sxnu.edu.cn (Y.L.); lijunru_1@163.com (J.L.); xssxsd2019@yeah.net (C.C.); l2419158655@163.com (H.L.); zhuzilin2002@163.com (Z.Z.); yan1743142072@126.com (Y.Y.); 19581558027@163.com (W.H.); 2 College of Medicine and Food Engineering, Shanxi University of Chinese Medicine, Taiyuan 030619, China

**Keywords:** naked oat peptides, ACE inhibition mechanisms, zinc tetrahedral coordination, molecular docking, security, stability

## Abstract

In this study, naked oat bran albumin hydrolysates (NOBAH) were subjected to gel chromatography with Sephadex G-15, reverse phase-high liquid performance separation, and UPLC-ESI-MS/MS identification. Six safe peptides including Gly-Thr-Thr-Gly-Gly-Met-Gly-Thr (GTTGGMGT), Gln-Tyr-Val-Pro-Phe (QYVPF), Gly-Ala-Ala-Ala-Ala-Leu-Val (GAAAALV), Gly-Tyr-His-Gly-His (GYHGH), Gly-Leu-Arg-Ala-Ala-Ala-Ala-Ala-Ala-Glu-Gly-Gly (GLRAAAAAAEGG), and Pro-Ser-Ser-Pro-Pro-Ser (PSSPPS) were identified. Next, in silico screening demonstrated that QYVPF and GYHGH had both angiotensin-I-converting enzyme (ACE) inhibition activity (IC_50_: 243.36 and 321.94 μmol/L, respectively) and Zinc-chelating ability (14.85 and 0.32 mg/g, respectively). The inhibition kinetics demonstrated that QYVPF and GYHGH were both uncompetitive inhibitors of ACE. Molecular docking showed that QYVPF and GYHGH could bind, respectively, three and five active residues of ACE with short hydrogen bonds (but not belonging to any central pocket). QYVPF and GYHGH could bind, respectively, twenty-two and eleven residues through hydrophobic interactions. Moreover, GYHGH was able to affect zinc tetrahedral coordination in ACE by interacting with His383. The inhibition activities of QYVPF and GYHGH toward ACE were relatively resistant to gastrointestinal digestion. GYHGH improved zinc solubility in the intestines (*p* > 0.05) because its amino and carboxyl groups were chelating sites for zinc ions. These results suggest the potential applications of naked oat peptides for potential antihypertension or zinc fortification.

## 1. Introduction

A quarter of the population worldwide suffers from hypertension, and approximately 10.8 million people die from hypertension complications every year [1]. To date, the key role of the ACE–Ang II–AT1R axis in hypertension has been well evidenced, and Angiotensin-I-Converting Enzyme (ACE) is one of the main targets of antihypertensive drugs [2]. ACE contains three active pockets including S1, S2, and S′ in the binding center, with zinc tetrahedral coordination in the catalytic center [3]. Inhibitors with binding affinity towards these pockets or zinc tetrahedral coordination should have a high inhibition capacity for ACE [4]. In recent years, an increasing number of studies showed that peptides with zinc-chelating abilities can inhibit ACE [3,5,6]. Moreover, zinc plays an important role in human health. Zinc deficiency mainly causes anorexia, slow growth, low immune function, and cognitive impairment [7]. Food-derived peptide−Zn chelates are considered ideal zinc supplement agents because they are more efficient, safe, and economical than inorganic zinc supplements such as zinc chloride and zinc sulfate [8,9]. Therefore, food-derived peptides that offer both antihypertensive effects and zinc fortification should be used more extensively in the food and medicine industries. In recent decades, natural antihypertensive peptides derived from foods have received increasing attention for their potential antihypertensive effects, economic benefits, and minor side effects [10,11]. However, few studies have simultaneously analyzed the inhibition activity on ACE and the zinc-chelating ability of peptides.

In addition to efficiency and bioavailability, other factors such as security, physicochemical properties, and stability can influence the applications of bioactive peptides in the healthcare, food, and pharmaceutical industries [12]. However, peptides with potential toxicity or allergenicity cannot be used in the food industry. Moreover, changes in the amino acid sequence, polarity, and electrochemical parameters of peptides can affect the interactions of peptides with ACE or zinc ions, leading to a decrease in the ACE-inhibitory activity, Zn-chelating ability, and stability of peptides [13,14]. Enzymes present in the gastric system or intestines can also hydrolyze peptides, leading to changes in the structure or physicochemical properties of the peptides [15].

Naked oat (*Avena nuda* L.) bran, a main byproduct of oat milling, is a good potential plant protein resource. This byproduct has high protein content (24.5–29.5 g/100 g), nearly equal to that of soybean and a well-balanced amino acid composition profile [16]. Another reason is the high annual yield of naked oat bran (approximately 850,000 tons in China) [17]. Albumin is one of the main protein fractions in naked oat bran, accounting for 19.72 g/100 g [18,19]. Our previous study showed that naked oat bran albumin hydrolysates (NOBAH) offered both ACE-inhibitory activity (23.63% ± 1.62%) and zinc-chelating ability (6.67± 0.13 mg/g), indicating that ACE-inhibitory peptides with high Zn-chelating capacity should be isolated from NOBAH. Although antioxidant peptides, antihypertensive peptides, and antibacterial peptides have been isolated from naked oat protein [20,21,22,23,24], simultaneous analysis of the inhibition activity toward ACE and the Zn-chelate capacity of naked oat peptides remains scarce. To fill this gap, in the present study, ACE-inhibitory peptides with Zn-chelating capacity were identified from NOBAH using in vitro assays combined with in silico tools. The inhibition mechanisms of NOBAH peptides toward ACE, particularly the interactions of the peptides with zinc tetrahedral coordination, were studied. The physicochemical characteristics, stability, potential toxicity, and allergenicity of NOBAH ACE-inhibitory peptides were also investigated.

## 2. Materials and Methods

### 2.1. Materials and Reagents

Naked oat bran was purchased from Sanfen Oat Farm, Shuozhou, China. Trypsin (1 × 10^4^ U/g, derived from porcine pancreases), Pepsin (1 × 10^5^ U/g, derived from bovine stomachs), Flavourzyme (from *Aspergillus oryzae*, 2 × 10^4^ U/g), pancreatin (1 × 10^4^ U/g, derived from porcine pancreases), and Papain (3 × 10^4^ U/g) were purchased from Kangfukuai Biotech., Co. (Nanning, China). ACE, 4-(2-Pyridinazo)-resorcinol (PAR) and *N*-hippuryl-L-histidyl-L-leucine (HHL) were purchased from Sigma (St. Louis, MO, USA). Zinc sulfate heptahydrate (ZnSO_4_·7H_2_O), HEPES-KOH buffer, and other analytical grade chemicals were purchased from Jinyangkeji Co. (Taiyuan, China).

### 2.2. Preparation of Naked Oat Bran Albumin Hydrolysates (NOBAH)

After grinding with a DYF-1000D grinder (Dade Machinery Factory, Taizhou, China), naked oat bran was filtered using a 100-mesh sieve (JL-007, Shangyu Instrument, Zhuji, China), and then defatted three times using petroleum ether (with a boiling range of 60–90 °C) with a mass to solvent ratio of 1:17. The defatted naked oat bran was suspended in deionized water (dH_2_O, 5 g/100 mL) and adjusted to pH 4.8. Next, cellulase (1.5 g/100 g naked oat bran) was added and stirred (in an XBZ-2 shaker, Yindu Instrument Co., Changsha, China) at 175 r/min and 50 °C for 180 min to further break down the cell walls and exclude starch [25]. The mixture was heated in boiling water for ten minutes to inactive the cellulase and then cooled and adjusted to pH 7.0. Then, the mixture was continuously stirred at 175 r/min and 40 °C for 4 h. After filtration on fast filter paper, the percolate was pooled and centrifuged at 14,000× *g* and 4 °C for 0.5 h to obtain the supernatant. The collected supernatant was sealed in a dialysis membrane (cut-off molecular weight of 5000 Da, Kesu Biotech., Co., Shanghai, China) and used for dialysis in dH_2_O at 4 °C. After 48 h dialysis, the residual solution in the dialysis bag was freeze-dried using a lyophilizer (LGJ-10N, Keya Inst., Co., Beijing, China) to obtain naked oat bran albumin (NOBA).

NOBA solution (2 g/100 mL dH_2_O) was adjusted to pH 7.5 using HCl (0.1 mol/L) or NaOH (0.1 mol/L) and mixed with Papain (75 mg) and Flavourzyme (75 mg). The digestion solution was shaken at 55 °C and 175 r/min for 95 min. Next, the digestion solution was heated in boiling water for 8 min and cooled to room temperature. After centrifugation at 12,000× *g* for 12 min, the supernatant was pooled and freeze-dried to obtain naked oat bran albumin hydrolysates (NOBAH). In addition, the trinitrobenzenesulfonic acid method was employed to determine the hydrolysis degree [26].

### 2.3. Isolation of Peptides with High Inhibition Ability on ACE

The NOBAH (1 mg/mL ultrapure water) was ultra-filtered using a W-45 membrane with a filter diameter of 0.45 μm (Jieneng Co., Wuxi, China) [24]. The filtrate was collected and lyophilized. The obtained powder was resolved in ultrapure water (1 mg/mL) and separated via gel chromatography on a column (Φ1.2 × 80 cm) filled with Sephadex G-15. The elution rate was 2.6 mL dH_2_O/min and monitored at 220 nm. The effluent fractions were collected and lyophilized to determine ACE-inhibitory activity. The subfraction with the highest ACE-inhibitory activity was further separated using reversed-phase high-performance liquid chromatography (RP-HPLC) on a Kromasil 100-5 C_18_ column (4.6 × 250 mm, 5 micron, Eka Chemicails, Sundsvall, Sweden) with a trifluoroacetate solution (1 mL/1000 mL ultrapure water) as elution solution A. Moreover, a linear gradient of acetonitrile containing 0.1% TFA (1–22%, in 15 min) was used as elution solution B. The flow velocity was maintained at 1.0 mL/min and the monitored wavenumber was 220 nm. The subfractions were separately collected and lyophilized, and their inhibition capacities toward ACE were measured. Among them, the subfraction that presented the highest inhibition capacity toward ACE was used for peptide sequence analysis.

### 2.4. Determination of Zn-Chelating Ability

NOBAH peptides (350 μg), 0.5 mL zinc sulfate solution (0.25 mmol/L), 0.5 mL DTT (8 mmol/L), 1 mL HEPES-KOH buffer (100 μmol/L), and 8 mL dH_2_O were mixed thoroughly [27]. After stirring at 37 °C and 175 r/min for 12 min, 1 mL of reaction solution was sucked out and used for zinc concentration determination using the 4-(2-Pyridinazo)-resorcinol method [8]. The standard regression curve of zinc concentration (*x*, μg/mL) with the absorbance at 500 nm (*y*) was *y* = 0.0901n(*x*) + 0.1012, R^2^ = 0.9802 [27]. The zinc chelation rate of the sample was defined as the reduction of zinc ions in the reaction solution per mass of the sample (mg/g).

### 2.5. Determination of ACE Inhibition Capacity and Inhibition Kinetics

Briefly, ACE (25 mU) was pre-incubated at 37 °C for 10 min. Then, ACE (75 µL), 225 µL of HHL (8.3 mmol/L), and peptide solution (75 µL) were mixed and stirred at 75 r/min and 37 °C for 60 min [28]. The reaction was stopped through the addition of 0.375 mL of HCl (1 mol/L). Next, 2.1 mL of ethyl acetate was added to extract the produced hippuric acid. After centrifugation at 14,000× *g* for 150 s, 1 mL of the upper solution (ethyl acetate extraction) was transferred into a glass test tube and heated at 120 °C for 32 min. Then, deionized water (1 mL) was added and determined at 228 nm. The control group was subjected to the same procedures but without samples. The inhibition ability of the samples toward ACE was defined as the percentage of the difference in absorbance at 228 nm between the sample and control compared to the absorbance of the control at 228 nm. The concentration of peptides needed to inhibit half of ACE activity was defined as IC_50_.

Moreover, the inhibition kinetics of samples on ACE were analyzed based on a Lineweaver–Burk plot of Angiotensin-I-Converting Enzyme with the addition of peptides (0–60 µmol/L) identified in QBGH, following the same procedures as Urbizo-Reyes et al. [11]. The substrate (HHL) concentration of ACE ranged from 0 to 7.60 mmol/L.

### 2.6. Identification, In Silico Screening, and Physicochemical Characterization of Peptide Sequences

Amino acid sequence identification was conducted on a Q Exactive hybrid quadrupole orbitrap mass spectrometer (Thermo Fisher, Bremen, Germany) according to the description of Li et al. [4]. Peak-Studio-7.5-De-Novo™ software (Bioinformatics Solutions, Inc., Waterloo, ON, Canada) was employed to analyze data from the mass spectrometer. The National Center for Biotechnology Information Database (Bethesda, MD, USA) was used for the verification of the obtained sequences. The PAR colorimetric method was employed to measure the Zn-chelating ability of the obtained sequences [8]. Potential antihypertensive effects were predicted using the database AHTPDB [29]. The predicted vector machine software score (SVMS) of antihypertensive peptides should be above zero [30]. Moreover, the physicochemical characteristics of the obtained peptides were analyzed with the database AHTPDB.

### 2.7. Chemical Synthesis

Chemical synthesis of the selected sequence identified in NOBAH was performed by Dingxiang Peptide Co. (Shaoxing, China) using the standard solid synthesis method [4].

### 2.8. Toxicity and Allergenicity Evaluation

The potential toxicity and allergenicity of NOBAH peptides were predicted with the databases ToxinPred and AlgPred, respectively [31].

### 2.9. Molecular Docking

An SYBLY-X.2.0.1 Murflex-Docking Tool (Tripos Int., Co., Saint Louis, MI, USA) was employed to visually simulate the coordination between the screened NOBAH peptides and the crystal structure of ACE [4]. The ACE structure with code PDB-108A downloaded from the Protein Data Bank (http://www.rcsb.org, accessed on 12 September 2022) was used as a docking template. The coordinated patterns of NOBAH peptides with ACE were chosen on the basis of the predicted total scores (T-scores, for which the acceptable threshold was 6.0), C-scores, and the number and length of hydrogen bonds. Moreover, the hydrophobic interactions of the NOBAH peptides screened with ACE were studied with LigPlot [28].

### 2.10. Interactions of Zinc Ions with NOBAH ACE-Inhibitory Peptides

#### 2.10.1. Preparation of the Peptide−Zn Complex

In a stirring water bath (175 r/min), the chemically synthesized NOBAH ACE-inhibitory peptides with the highest ACE-inhibitory activity (200 μg) were dispersed and reacted with 5 mmol/L of ZnSO_4_·7H_2_O (1.4 mL) at pH 6.2 and 63 °C for 55 min [27]. After centrifugation at 4500× *g* for 35 min, the supernatant was mixed with four times the volume of anhydrous ethanol, and then placed at room temperature for 35 min. Then, the mixed solution was centrifuged at 12,500× *g* for 8 min. The precipitate was gathered and washed using anhydrous ethanol three times and then lyophilized to obtain NOBAH peptides−Zn complexes.

#### 2.10.2. Coordination Patterns of NOBAH Peptides with Zinc Ions

The coordination patterns of NOBAH peptides and zinc ions were analyzed with Fourier-transformed infrared spectroscopy (FT-IR) [9]. Briefly, the NOBAH peptide−Zn complexes’ powder (2 mg) and 100 mg of dry KBr were mixed, ground, pelleted, and loaded on an FT-IR spectrometer (660-IR, Varian, Palo Alto, CA, USA). The scanning range was 4000 to 400 cm^–1^ with a resolution of 4 cm^–1^. NOBAH peptides were used as a comparison.

### 2.11. Stabilities of NOBAH Peptides

The simulation intestinal digest mucus (pH 6.80 ± 0.10) was composed of 6 g bile salt, 0.07 g pancreatin, 12.5 g NaHCO_3_, and 200 mL ultrapure water. The simulation gastric digest mucus (pH 2.00 ± 0.10) contained 0.15 mol/L of NaCl and 0.35 mg/mL of pepsin [14]. NOBAH peptides (5 g) were first hydrolyzed with the simulation gastric digest mucus (30 mL). The digest was stirred at 135 r/min and 37 °C for 80 min. Next, the pH value of the digest was adjusted to pH 6.8 and 50 mL of the simulated intestinal digest mucus was added. After stirring at 135 r/min and 37 °C for 150 min, the digest was placed at 100 °C for 10 min. The ACE inhibition activity of NOBAH peptides was then determined with untreated NOBAH peptides as the comparison.

Simultaneously, 5 mg of NOBAH peptide−Zn complexes were mixed with 50 mL of simulation gastric digest mucus (pH 2.00 ± 0.10) [6]. After shaking at 37 °C and 135 r/min for 90 min, Na_2_HPO_4_ (0.5 mol/L) was added and quickly stirred until the pH value of the digest was increased to 6.80 ± 0.10. Then, the simulated intestinal digest mucus (50 mL) was added. The digest was continuously shaken at 37 °C and 135 r/min for 150 min. At every 0.5 h interval, an aliquot of the digest (0.8 mL) was taken out for zinc concentration determination via the 4-(2-Pyridinazo)-resorcinol method [8]. The stability of NOBAH peptide−Zn complexes was expressed as the residual zinc concentration after digestion as a percentage of the zinc concentration before digestion. Zinc gluconate (100 μg/mL) and ZnSO_4_ (100 μg/mL) were used as comparisons.

### 2.12. Data Analysis

The results of all tests were expressed as the mean ± standard deviation (the number of experimental repetitions was at least 3). The difference between data was analyzed using a one-way analysis of variance coupled with Duncan’s multiple tests using the IBM SPSS Statistics software (Version 16, Chicago, IL, USA). The difference was accepted as significant at *p* < 0.05.

## 3. Results and Discussion

### 3.1. Selection of Fractions with High ACE-Inhibitory Activity from NOBAH

**Figure 1:**
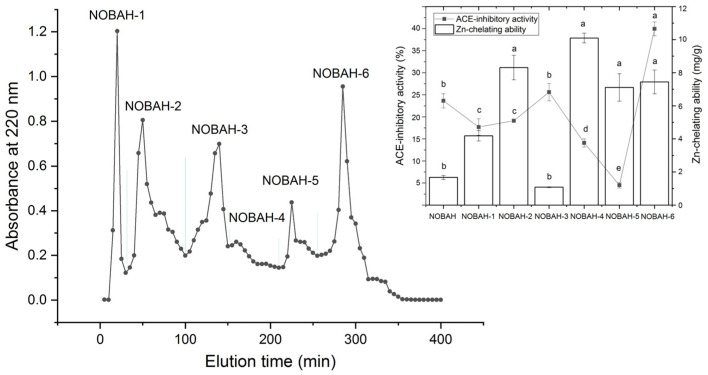
The purification spectra of naked oat albumin hydrolysates (NOBAH) using gel chromatography with Sephadex G-15, and the inhibition activity on ACE and zinc-chelating capacity of the six obtained subfractions NOBAH-1, NOBAH-2, NOBAH-3, NOBAH-4, NOBAH-5, and NOBAH-6. Different lowercase letters (a–e) above the bars or near the line mean that the difference is significant (*p* < 0.05).

In this study, the hydrolysis degree of naked oat bran albumin using Papain and Alcalase was found to be 27.62% ± 3.77%, which was lower than that of oat protein isolates digested using the mix enzymes of Alcalase, Flavourzyme, Papain, and Protamex (69.9%) [20]. Flavourzyme and Papain are widely used for the preparation of antihypertensive peptides because they have both protease and esterase activities with broad specificity [32,33]. The ACE-inhibitory activity of NOBAH was 23.63% ± 1.62% (1 mg/mL), which was lower than that of naked oat globulin hydrolysates (48.18% ± 4.02%) [24]. However, the Zn-chelating ability of NOBAH (1.67 ± 0.13 mg/g, Figure 1) was much higher than that of naked oat globulin and glutelin (0.22 ± 0.02 mg/g). Previous studies showed naked oat albumin to be rich in Glu, Asp, His, and Gly, while the content of these amino acids in naked oat globulin was found to be relatively lower [17,18]. The γ-carboxyl group and ε-amino group in Glu or His have a high chelating ability with metal ions [34], which was mainly responsible for the high Zn-chelating ability of NOBAH.

**Figure 2:**
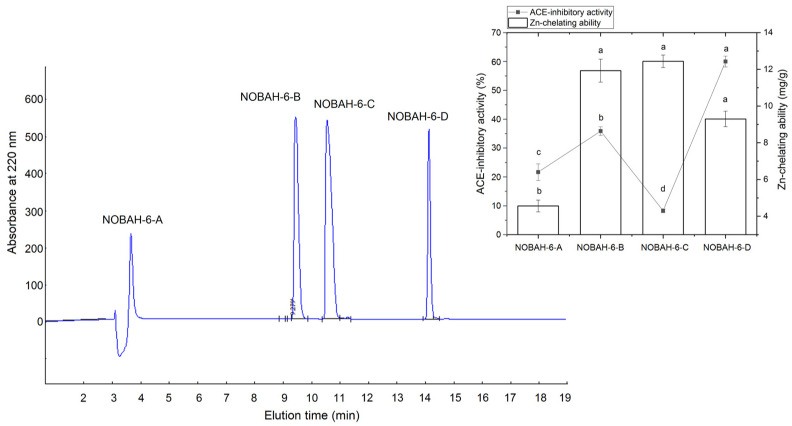
The reverse-phase high-performance liquid chromatographic profiles of the subfraction NOAH-6 and the inhibition activity toward ACE and zinc-chelating capacity of the four subfractions NOBAH-6-A, NOBAH-6-B, NOBAH-6-C, and NOBAH-6-D. Different lowercase letters (a–d) on the bars or near the line mean that the difference is significant (*p* < 0.05).

The profile in Figure 1 indicates that NOBAH was divided into six major subfractions (NOBAH-1, NOBAH-2, NOBAH-3, NOBAH-4, NOBAH-5, and NOBAH-6) after purification via G-15 gel chromatography. Among these subfractions, NOBAH-6 exhibited the highest inhibition activity toward ACE (39.95% ± 1.57% at 1 mg/mL, Figure 1) and high zinc-chelating capacity (7.44 mg/g). Thus NOBAH-6 was further separated with RP-HPLC on an analytical C_18_ column, and four major subfractions were obtained (Figure 2). The ACE-inhibitory activities of NOBAH-6-A, NOBAH-6-B, NOBAH-6-C, and NOBAH-6-D were 21.65% ± 2.87%, 25.86% ± 1.45%, 8.28% ± 0.68%, and 59.97% ± 1.86% mg/g (at 1 mg/mL), respectively. In addition, NOBAH-6-D exhibited a strong Zn-chelating activity (9.30 ± 0.42 mg/g). Therefore, NOBAH-6-D was used for the identification of amino acid sequences.

### 3.2. Identification and Characterization of Peptides from NOBAH-4-D

As shown in Table 1, six peptides, Gly-Thr-Thr-Gly-Gly-Met-Gly-Thr (GTTGGMGT), Gln-Tyr-Val-Pro-Phe (QYVPF), Gly-Ala-Ala-Ala-Ala-Leu-Val (GAAAALV), Gly-Leu-Arg-Ala-Ala-Ala-Ala-Ala-Ala-Glu-Gly-Gly (GLRAAAAAAEGG), Gly-Tyr-His-Gly-His (GYHGH), and Pro-Ser-Ser-Pro-Pro-Ser (PSSPPS) were identified in NOBAH-6-D on the basis of the UPLC-ESI-MS/MS analysis results. In this study, the potential antihypertension of these peptides was accepted if their vector machine software score was above zero [3]. Both GYHGH and QYVPF showed potential antihypertensive properties because their vector machine software scores (0.90 and 1.21, respectively) were greater than zero [4,30].

**Figure 3:**
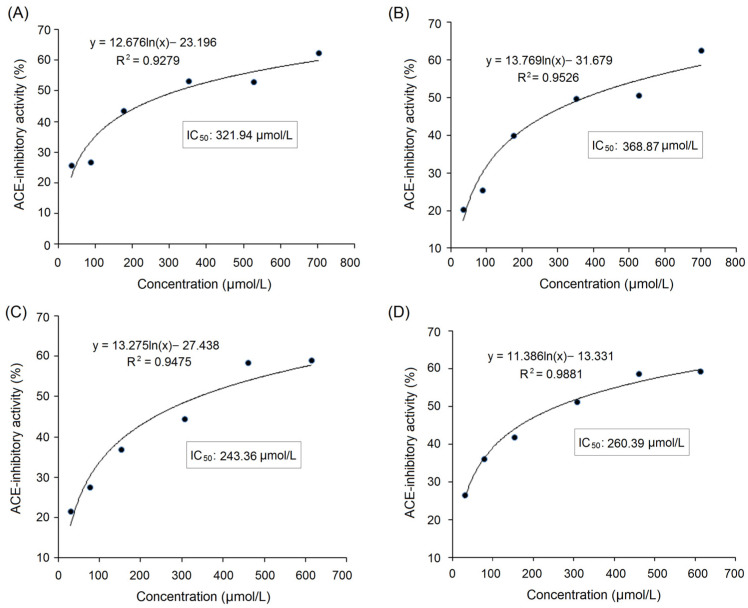
The regression analysis on the inhibition capacity of peptides GYHGH (**A**) and QYVPF (**C**) toward ACE and a regression analysis on the inhibition capacity of GYHGH (**B**) and QYVPF (**D**) against ACE after simulated gastrointestinal digestion. IC_50_ value is the concentration of peptides when the inhibition activity toward ACE was 50%.

As shown in Figure 3A,C, the relationship between the inhibition abilities of synthesized GYHGH and QYVPF toward ACE (*y*) with concentration (*x*) were *y* = 12.676ln(*x*) − 23.196, and *y* = 13.275ln(*x*) − 27.438, respectively. The IC_50_ values of GYHGH and QYVPF were 321.94 and 243.36 μmol/L, respectively, verifying the in silico prediction results with the AHTPDB database (Table 1). The inhibition activities of GYHGH and QYVPF toward ACE were higher than those of peptides with similar mass values, such as QPHQPL identified in rubing cheese (IC_50_: 464.20 μmol/L), but lower than those of LFRPE from *Boletus griseus* (IC_50_: 11.34 μmol/L) and Captopril (an excellent antihypertensive drug with an IC_50_ value of 0.14 μmol/L) (*p <* 0.05) [4,35]. The inhibition capacities of GYHGH and QYVPF toward ACE were also higher than those of peptides ELHPQ and SVPGCT isolated from canary seed protein and lupin protein hydrolysates, respectively (*p* < 0.05) [6,33]. These results suggest that GYHGH and QYVPF offer relatively high ACE-inhibitory activities.

It was shown that the hydrophobic amino acids in the *C*-terminal tripeptide, especially the Phe, Tyr, Trp (belonging to aromatic amino acids), Leu, Ile, Val (branched amino acids), and Pro residues, are crucial for the inhibition effects of peptides against ACE, as these hydrophobic amino acids have a relatively high binding affinity with the active sites of ACE [36]. Moreover, Lys and His residues in the *C*-terminal tripeptide are instrumental in the ACE-inhibition ability of peptides [11]. Previous studies also found that the Tyr residue near the *N*-terminal can facilitate the coordination of peptides with ACE [28]. Therefore, amino acid residues, especially the Pro, Phe, His, Val, and Tyr residues present in the *C*-terminal tripeptide or near the *N*-terminal, contributed most to the high inhibitory capacities of GYHGH and QYVPF against ACE.

**Figure 4:**
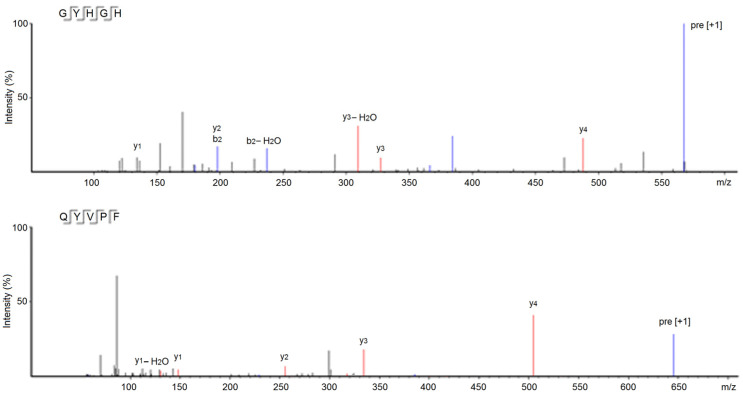
ESI-MS/MS spectra of peptides GYHGH and QYVPF identified in naked oat albumin hydrolysates.

In addition, GYHGH showed the highest Zn-chelating ability (14.85 ± 0.39 mg/g), in accordance with its high basic amino acid content (40%, Table 1). The other five peptides including GTTGGMGT, GLRAAAAAAEGG, and QYVPF also showed considerable Zn-chelating abilities (0.32–4.95 mg/g, Table 1). Research on the structure-activity relationship of peptide-zinc chelates showed that the amino and carbonyl groups of peptides were ideal zinc-binding sites [6]. The nitrogen atoms of the second and third amide bonds at the *N*-terminal can take part in coordination with zinc ions [8]. Glu or Asp with the γ-carboxyl group, and His or Lys with the ε-amino group, can all increase the negative polarity of peptides and improve the link force of peptides with zinc ions. Additionally, Gly and Pro residues can be used as a negative bridge ligand for zinc ions [37]. Hence, amino acid residues, especially His, Pro, and Gly residues, are predominantly responsible for the high binding affinity of GYHGH with zinc ions. Figure 4 shows the ESI-MS/MS spectra of GYHGH and QYVPF, from which their amino acid sequences and molecular weights were obtained.

### 3.3. Physicochemical Characterization In Silico

As shown in Table 1, QYVPF had a high content of hydrophobic amino acids (60.00%) and relatively high hydrophobicity (0.08), which was another reason for its high inhibition capacity toward ACE, as hydrophobic residues were found to increase the link force of peptides towards ACE active sites [38]. Moreover, GYHGH exhibited the highest amphiphilicity (1.59) and considerable hydrophilicity (−0.09), corresponding to its high Zn-chelating ability (17.30 ± 1.46 mg/g), because hydrophilic groups of peptides have a greater polar charge for binding with zinc ions [36]. Additionally, the isoelectric points (pI) of GYHGH and QYVPF were 7.25 and 5.88, respectively. Peptides should avoid being used in solutions with pH values near their isoelectric points. At isoelectric points, the polarity and solubility of peptides are dramatically deceased, leading to adverse effects on the coordination of peptides with ACE or zinc ions [27].

### 3.4. Security Predictions In Silico

As shown in Table 1, the in silico results predicted with the ToxinPred database demonstrated that GTTGGMGT, QYVPF, GAAAALV, GLRAAAAAAEGG, GYHGH, and PSSPPS were all non-toxic peptides. These peptides, moreover, did not match any allergic peptides that were recorded in the AlgPred database. In addition, short oligopeptides are less likely to have complete epitopes than proteins with a larger mass [39]. These results indicate that these four peptides have no potential allergenicity. However, further security studies, including cellular tests and in vivo assays, are still needed.

### 3.5. Inhibition Mechanisms on ACE

#### 3.5.1. Molecular Docking

**Figure 5:**
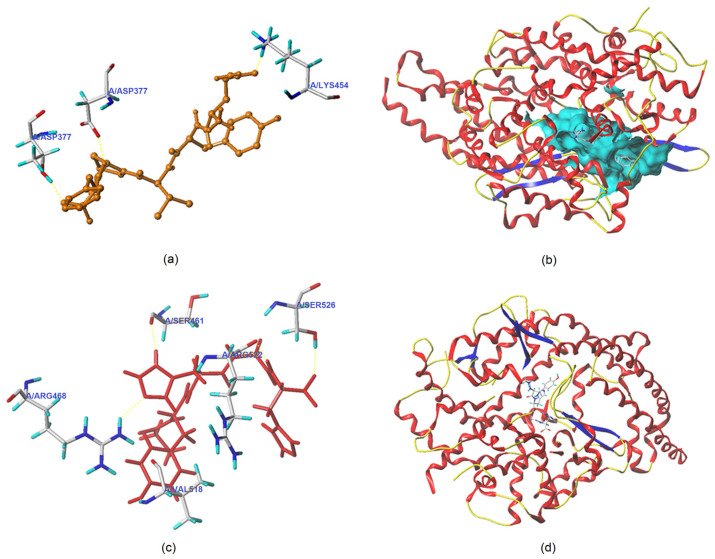
Three-dimensional images showing a local perspective of the best-ranked docking modes of QYVPF (**a**) and GYHGH (**c**) with ACE (PDB: 1O8A) and a general overview of the best docking modes of QYVPF (**b**) and GYHGH (**d**) with ACE.

Figure 5a–d depicts the best docking modes of QYVPF and GYHGH with ACE (PDB: 1O8A) from a local and general perspective, respectively. As shown in Figure 5a, QYVPF and three active sites of ACE (Asp377, Thr372, and Lys454) can be linked by short hydrogen bonds. GYHGH can bind with five active residues of ACE including Arg468, Ser526, Arg522, Val518, and Ser461 through short hydrogen bonds (Figure 5c). Hydrogen bonds are instrumental in the coordination of peptides with ACE.^4^ The hydrogen bond distance of QYVPF and GYHGH with ACE was found to be short (1.98–2.93 Å). In addition, the total scores (T-scores) of QYVPF and GYHGH with ACE (8.95 and 8.04, respectively) were higher than the acceptable threshold (6.0) [30]. The T-score can reflect the affinity of peptides with ACE, which is dependent on hydrogen bonds, Van der Waals forces, and hydrophobic interactions [4]. In addition, QYVPF and GYHGH were found to bind with twenty-two and eleven residues through hydrophobic interactions, respectively (Table 2). These results indicate a strong affinity of QYVPF or GYHGH towards ACE, corresponding to their relatively high ACE-inhibitory activities (IC_50_: 243.36 and 321.94 μmol/L, respectively, as shown in Table 1). However, the active sites formed between ACE and QYVPF or GYHGH did not belong to any active pocket of ACE, suggesting that QYVPF and GYHGH are uncompetitive inhibitors of ACE. For this reason, the ACE-inhibitory activities of QYVPF and GYHGH were lower than those of LFRPE identified in *Boletus griseus* (a competitive inhibitor) [4].

Moreover, GYHGH can interact with His383, which belongs to the zinc tetrahedral coordination of ACE through hydrophobic interactions (Table 2). Zinc tetrahedral coordination, through which a zinc ion coordinates with residues His387, Glu411, and His383, occurs in the catalytic center of ACE and is crucial for the catalytic action of ACE [2]. The interactions of GYHGH with His383 indicated that GYHGH can inhibit ACE by affecting zinc tetrahedral coordination. Oligopeptides identified in *Lepidium* and *Boletus griseus*, including LFRPE, RSRGVFF, and KYPHVF, were also found to impact zinc tetrahedral coordination by binding with His383 in ACE and presented strong ACE-inhibitory activities [4,40].

#### 3.5.2. Inhibition Kinetics on ACE

**Figure 6:**
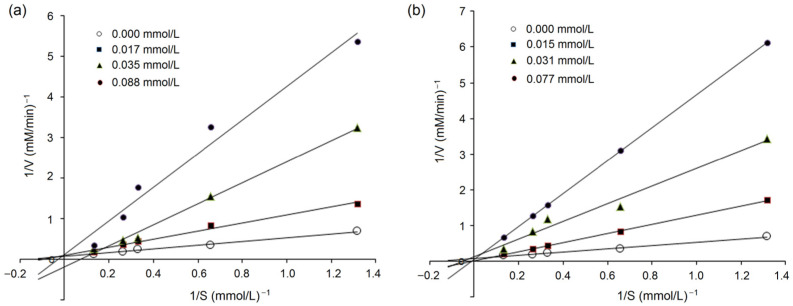
Lineweaver–Burk plots showing ACE inhibition of GYHGH (**a**) and QYVPF (**b**).

Lineweaver–Burk plots of ACE against various concentrations of HHL with peptides QYVPF and GYHGH are shown in Figure 6a and Figure 6b, respectively. The kinetic constants demonstrated that *K_m_* increased as the concentration of QYVPF or GYHGH increased, whereas the V_max_ (the maximum velocity) of the reaction decreased. These results suggest that QYVPF and GYHGH were both uncompetitive inhibitors of ACE, consistent with the results of molecular docking (Figure 5 and Table 2).

#### 3.5.3. Coordination Patterns of QYVPF and GYHGH with Zinc Ions

**Figure 7:**
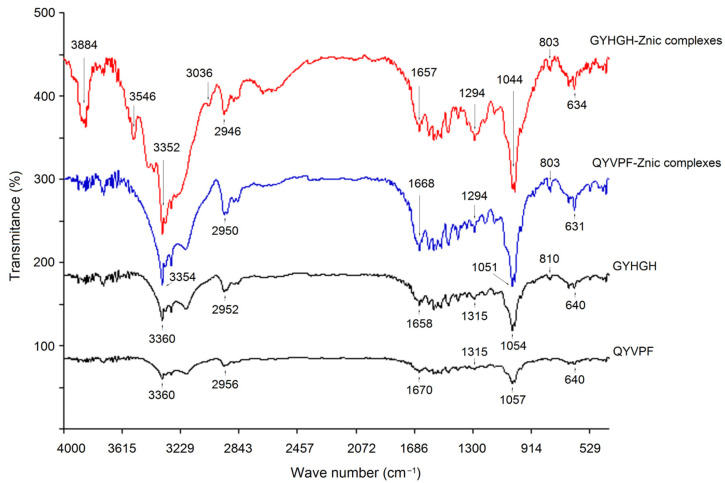
Fourier-transformed infrared spectra of the GYHGH−Zn complexes, QYVPF−Zn complexes, pure GYHGH, and QYVPF.

The coordination patterns of QYVPF and GYHGH with zinc ions were further studied via FT-IR, and the results are shown in Figure 7.

Significant differences were observed between the FT-IR spectra of GYHGH−Zn complexes and those of GYHGH. The peak at 3360 cm^−1^ in the infrared spectrum of GYHGH was indicative of the deformation of –N–H [37], while in the infrared spectrum of GYHGH−Zn complexes, this peak appeared at 3352 cm^−1^. Moreover, the peak at 1658 cm^−1^ in the spectrum of GYHGH representing the stretching vibrations of –C–N bonds transferred to 1657 cm^−1^ after Zn-chelation [34]. After Zn-chelation, a red-shift appeared at peaks of 640 and 810 cm^−1^ in the FT-IR spectra of GYHGH−Zn chelates, which all corresponded to the stretching of the amide IV band. These results demonstrated that the amino groups and amide bonds of GYHGH chelated with zinc ions [6]. Additionally, the peak at 1315 cm^−1^ in the spectrum of GYHGH representing the stretching vibration of the –C–O bond transferred to 1294 cm^−1^ after Zn-chelation, suggesting the formation of –COO–Zn [8]. A similar difference was observed in the spectra of QYVPF−Zn complexes between the spectra of QYVPF. Therefore, the carboxyl groups, ε-amino groups, and amide bonds of QYVPF and GYHGH can all chelate with zinc ions.

### 3.6. Stability against Gastrointestinal Digestion

As shown in Figure 3B,D, based on regression equations between the ACE inhibition abilities (*y*) of QYVPF and GYHGH with their concentrations (*x*), the IC_50_ values of QYVPF and GYHGH against ACE were calculated as 260.39 and 368.87 μmol/L after gastrointestinal digestion, which was not significantly different from that of the untreated QYVPF and GYHGH (243.36 and 321.94 μmol/L, respectively, as shown in Table 1) (*p* > 0.05), indicating that the inhibition abilities of QYVPF and GYHGH toward ACE were relatively stable under gastrointestinal hydrolysis.

**Figure 8:**
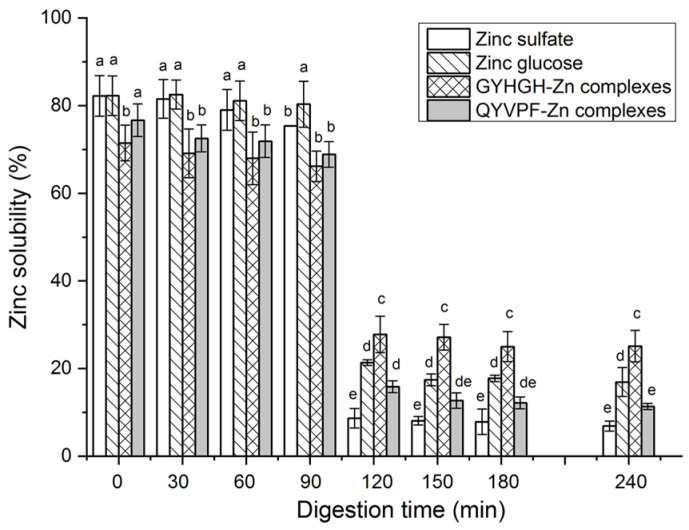
Zinc solubility of zinc sulfate, zinc gluconate, and QYVPF− and GYHGH−Zn complexes against simulated gastrointestinal digestion. Different lowercase letters (a–e) on the bars indicate that the difference is significant (*p* < 0.05).

Moreover, as shown in Figure 8, during digestion with gastric digestive mucus (0–90 min), both QYVPF−Zn complexes and GYHGH−Zn complexes exhibited relatively stable Zn solubility. However, their Zn solubility dramatically decreased during digestion with intestinal digestive mucus (90–240 min) (*p <* 0.05). ZnSO_4_ and zinc gluconate both showed a similar trend. The main reason for this result is that from the stomach to the intestines, the pH of digestive fluid dramatically rises from 2.0 to 7.0, and most zinc ions in the digestive fluid form insoluble zinc salts as pH increases [37]. From 120 to 240 min, both GYHGH−Zn complexes and zinc gluconate showed higher zinc solubility than that of zinc sulfate (*p* < 0.05), evidencing that GYHGH can improve zinc stability in the intestines. In peptide-zinc complexes, zinc ions are generally located within the peptide chain structure, protecting zinc ions from pH interference [5]. In comparison, the zinc solubility of GYHGH−Zn complexes was not significantly different from that of zinc sulfate (*p* > 0.05), corresponding to its low Zn-chelating ability (0.32 ± 0.09 mg/g, Table 1).

It was shown that enzymes existing in the stomach or intestines can cleave polypeptides, especially peptide chains with Phe, Tyr, Lys, and Arg residues, resulting in decreased bioactivities of peptides [32]. Although QYVPF and GYHGH both contain a Tyr residue, they exhibited relatively stable ACE-inhibitory activity and Zn-chelating capabilities under gastrointestinal hydrolysis, which is likely attributable to Pro and His residues in QYVPF and GYHGH. Pro and His both have rigid ring structures that can improve the stability of peptides against gastrointestinal enzymes [6,28]. However, further studies are needed to analyze the effects of gastrointestinal hydrolysis on the structures of QYVPF and GYHGH.

## 4. Conclusions

Two uncompetitive ACE inhibitors with Zn-chelating capacity, QYVPF and GYHGH, were identified in NOBAH. Molecular docking showed that QYVPF and GYHGH could bind with three and five active residues of ACE with short hydrogen bonds, respectively, but did not belong to any central pocket. QYVPF and GYHGH were found to bind with twenty-two and eleven residues, respectively, through hydrophobic interactions. Moreover, GYHGH was able to affect zinc tetrahedral coordination in ACE by interacting with His383. The inhibition capacities of QYVPF and GYHGH towards ACE were resistant to gastrointestinal digestion. GYHGH improved zinc solubility in the intestines (*p >* 0.05). These results suggest that naked oat peptides can be used as ingredients in antihypertensive agents or zinc supplements.

## Figures and Tables

**Table 1:** Amino acid sequences, ACE-inhibitory activity, Zn-chelating capacity, physicochemical characteristics, toxicity, and allergenicity of peptides isolated from naked oat bran albumin hydrolysates.

Peptide Sequence	GTTGGMGT	GYHGH	GLRAAAAAAEGG	QYVPF	GAAAALV	PSSPPS
Mass (Da)	579.73	569.65	1014.25	652.81	571.75	570.66
Matched sequence in *Avena nuda* ^a^	H.GTTGGMG.T	G.GYHGH.G	G.GLRAAAAAAEGG.M	Q.QYVPF.A	L.GAAAALV.F	-.PSSPPS.V
SVMS ^b^	−0.24	0.90	−0.50	1.21	−0.94	−0.31
Antihypertension prediction	Non-AHT	AHT	Non-AHT	AHT	Non-AHT	Non-AHT
ACE-inhibitory activity (IC_50_: μmol/L)	ND	321.94	ND	243.36	ND	ND
Zinc chelating capacity (mg/g)	3.36 ± 0.16 e	14.85 ± 0.39 d	4.95 ± 0.07 e	0.32 ± 0.09 g	1.27 ± 0.22 ef	0.42 ± 0.06 g
Basic or acidic amino acid content (%)	0.00%	40.00%	16.67%	0.00%	0.00%	0.00%
Hydrophobic amino acid content (%)	12.50%	0.00%	50.00%	60.00%	85.71%	50.00%
Physicochemical properties						
Hydrophobicity	0.08	−0.09	0.01	0.08	0.32	−0.17
Amphiphilicity	0.00	1.59	0.31	1.26	0.00	0.00
Hydrophilicity	−0.30	−0.66	0.10	−1.22	−0.76	0.15
Isoelectric point	5.88	7.25	6.36	5.88	5.88	5.88
Security ^c^						
Toxicity	Non-Toxin	Non-Toxin	Non-Toxin	Non-Toxin	Non-Toxin	Non-Toxin
Allergenicity	No	No	No	No	No	No

^a^ From the National Center for Biotechnology Information (NCBI); ^b^ SVMS (vector machine software score) and physicochemical characteristics were in silico analyzed by the AHTPDB database; AHT: antihypertension; ^c^ Safety including toxicity and allergenicity were analyzed with assistance from the databases ToxinPred and AlgPred, respectively. ND: not measured. Different lowercase letters (d–g) in the same line mean that the difference is significant (*p* < 0.05).

**Table 2:** Interactions of GCHHY and QYVPF with the active sites of ACE from molecular docking simulation.

Ligand	T-Score	C-Score	Interaction Mode	ACE Residues and the Length of Hydrogen Bonds Formed between ACE and Ligand
QYVPF	8.95	4	Hydrogen bond	Thr372: 2.60Å; Asp377: 2.85Å; Lys454: 2.93Å
			Hydrophobic interaction	Glu162, Pro163, Gln369, Cys370, Trp279, Cys352, His353, Val380, Ala354, Lys511, Tyr520, Asp415, Val379, Phe527, Tyr523, Phe457, Gln281, Thr166, Glu376, Asn374, Ala170, Asn167
GYHGH	8.04	3	Hydrogen bond	Arg468: 2.02Å; Ser526: 2.14Å; Arg522: 1.98Å; Val518: 2.07Å; Ser461: 2.08Å
			Hydrophobic interaction	Gln369, Asp377, Ser355, His383, Tyr520, Phe457, Val380, Val379, Trp279, Asn277, Thr166

## Data Availability

The data that support the findings of this study are available from the corresponding author upon reasonable request.
